# The Dual Role of Nrf2 Signaling in Virus Infections: Antiviral Guardian or Proviral Accomplice?

**DOI:** 10.3390/pathogens15010008

**Published:** 2025-12-20

**Authors:** Bikash R. Sahoo, Kush K. Pandey, Asit K. Pattnaik

**Affiliations:** 1 School of Veterinary Medicine and Biomedical Sciences, University of Nebraska-Lincoln, Lincoln, NE 68583, USA; bsahoo2@unl.edu (B.R.S.); kpandey2@huskers.unl.edu (K.K.P.); 2 Nebraska Center for Virology, University of Nebraska-Lincoln, Lincoln, NE 68583, USA

**Keywords:** Nrf2, Keap1, oxidative stress, virus infections, antiviral immunity

## Abstract

The transcription factor nuclear factor erythroid 2-related factor 2 (Nrf2) plays a critical role in regulating cellular defense against oxidative stress and maintaining redox homeostasis. In the context of viral infections, Nrf2 signaling emerges as a double-edged sword. On one hand, it activates a broad spectrum of antioxidant and cytoprotective genes, contributing to host defense and antiviral immunity. On the other hand, certain viruses exploit the Nrf2 pathway to create a favorable environment for replication, persistence, or immune evasion. This review summarizes the current understanding of Nrf2’s antiviral and proviral roles in both RNA and DNA virus infections, delineates the underlying mechanisms, and discusses the therapeutic implications of targeting Nrf2. We emphasize the need for context-dependent modulation of Nrf2 activity and highlight future directions in precision antiviral strategies.

## 1. Introduction

Oxidative stress, a hallmark of viral infections, plays a crucial role in determining host–pathogen interactions. Reactive oxygen species (ROS), such as superoxides, hydroxyl radicals, and hydrogen peroxide generated by mitochondrial dysfunction, NADPH oxidases, and other cellular sources, induce oxidative stress and have been shown to serve both anti- and proviral purposes during viral infection [1,2,3,4]. ROS can act as signaling molecules and aid in orchestrating innate immune responses and antiviral defenses. Reactive nitrogen species (RNS) such as peroxinitrite and nitrogen dioxide generated through interactions of superoxides with nitric oxide are also known to induce oxidative stress. Collectively, reactive oxygen and nitrogen species (RONS) at low levels are essential for normal cell growth and proliferation, tissue repair, angiogenesis, and maintenance of cellular homeostasis [5,6,7], but excessive or prolonged oxidative stress can damage lipids, proteins, and nucleic acids, thereby compromising cellular integrity and facilitating many disease conditions [1,2,8]. As a result, the cellular response to oxidative stress is tightly regulated, with nuclear factor erythroid 2-related factor 2 (Nrf2) playing a central role in maintaining redox homeostasis and protecting against oxidative damage [3,9,10].

Nrf2 was first recognized as a transcription factor for its role in chemoprevention [11,12] but studies in the past three decades or so have revealed that Nrf2 is a master regulator of not only the antioxidant response to maintain redox balance but also for other cellular processes including proteostasis, metabolic stability, and intracellular iron dynamics, which are critical for the cellular response to adverse environmental and metabolic conditions [6,7,13,14,15]. The cloning of Nrf2 in the mid-90s [16,17] led to an explosion of studies on the characterization and regulation of Nrf2. These studies revealed that Nrf2 is a cap-and-collar subfamily of the basic leucine zipper-type (CNC-bZIP) family of transcription factors, which is encoded by the nuclear factor, erythroid-derived 2-like 2 (*NFE2L2*) gene [16,17]. Under basal conditions, the Nrf2 mRNA is constitutively transcribed and translated to produce the Nrf2 protein, but levels of the protein remain low due to proteolytic degradation in the cytoplasm through specific interactions with its negative regulator, Kelch-like ECH-associated protein 1 (Keap1), which targets Nrf2 for ubiquitin-mediated proteasomal degradation [18,19,20,21,22] (see below for more detailed description of Nrf2 degradation pathway). However, upon oxidative or electrophilic stress, modifications in Keap1 lead to its dissociation from Nrf2, resulting in stabilization and nuclear translocation of Nrf2. In the nucleus, Nrf2 binds to antioxidant response elements (AREs) in the promoters of target genes, driving the expression of cytoprotective enzymes and proteins. These include heme oxygenase-1 (HO-1), NAD(P)H quinone dehydrogenase 1 (NQO1), glutamate-cysteine ligase (GCL), and glutathione S-transferases (GSTs). Through these cellular proteins, Nrf2 promotes detoxification, glutathione synthesis, redox balance, and inflammation resolution [3,9]. Importantly, numerous RNA and DNA viruses interact with and modulate redox signaling pathways during infection. Viral replication often leads to increased ROS production, either as a byproduct of hijacked cellular metabolism or as a deliberate strategy to bypass host defenses [1]. In response, the Nrf2 pathway is activated as a countermeasure. Emerging evidence suggests that viruses have evolved sophisticated strategies to manipulate Nrf2 signaling to their advantage. For instance, some viruses activate Nrf2 to protect infected cells from oxidative damage and promote cell survival, thereby enhancing viral replication or persistence. Conversely, others suppress Nrf2 activity to exacerbate oxidative stress and inflammation, which may aid in viral dissemination or immune evasion. Consequently, the role of Nrf2 in viral infections is highly context-dependent, with both antiviral and proviral consequences [9].

In RNA virus infections, Nrf2 activation has been demonstrated to restrict viral replication and reduce inflammation in specific contexts, such as in the influenza A virus and SARS-CoV-2 infections. Given their ability to limit oxidative tissue damage and modulate the inflammatory response, Nrf2 inducers have been proposed as therapeutic agents in these settings. Conversely, during infections with other viruses such as the hepatitis C virus (HCV) and respiratory syncytial virus (RSV), Nrf2 activity may be co-opted by the virus to support replication, suggesting that Nrf2 activation can also contribute to chronicity and persistence. Similar complexity exists with DNA virus infections as well, where African swine fever virus (ASFV) [23], vaccinia virus [24], and Kaposi’s sarcoma-associated herpesvirus (KSHV) [25,26] have all been shown to engage Nrf2 signaling, sometimes to promote replication, latency, transformation, or resistance to cell death. This intricate interplay between Nrf2 signaling and viral infection underscores the broader significance of redox signaling in host–pathogen dynamics. Beyond Nrf2, redox-sensitive pathways influence various cellular processes, including viral sensing, inflammation, apoptosis, and autophagy. Understanding how viruses manipulate these redox circuits is pivotal in deciphering their pathogenesis and developing host-directed therapeutic interventions.

In this review, we provide an overview of the current understanding of Nrf2 and redox signaling in the context of viral infections. We delve into the mechanisms by which viral infections modulate Nrf2 signaling, explore the downstream consequences for viral replication and host responses, and highlight the dual roles of Nrf2 as both a pro- and antiviral factor in the context of RNA and DNA viruses. Furthermore, we discuss the therapeutic implications of targeting Nrf2 in viral diseases, emphasizing the importance of context-specific modulation strategies.

## 2. Nrf2 Signaling Pathways and Regulation: Key Players and Mechanisms of Nrf2 Activation and Suppression

As described above, Nrf2 is constitutively expressed but is maintained at basal levels, which is essential for cellular homeostasis. Upon induction of oxidative and/or electrophilic stress, Nrf2 is activated through a complex network of regulatory steps that allows increased accumulation and nuclear translocation of the transcription factor, which then orchestrates transcriptional activation of antioxidant response genes. The activity and levels of Nrf2 are regulated by multiple signaling pathways that include the canonical Keap1 cysteine residues-dependent pathway and non-canonical Keap1 cysteine-independent pathway involving the autophagy receptor p62/sequestosome 1(SQSTM1) [6]. In the following sections, we describe how Nrf2, Keap1, and p62 activities are regulated under basal and oxidative stress conditions and the underlying mechanisms.

### 2.1. Nrf2

**Figure 1:**
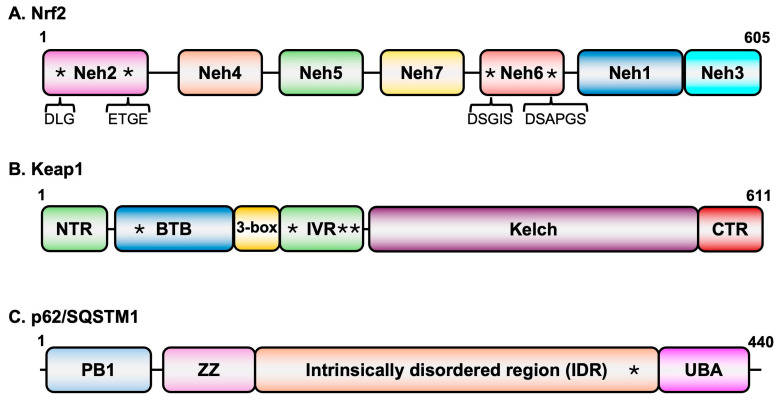
Domain structures of key proteins in oxidative stress signaling. (**A**) Nrf2 domain structure. Asterisks in Neh2 domain represent the two conserved sequence motifs that interact with Keap1 while those in the Neh6 domain represent motifs that are recognized by E3 ubiquitin ligase b-TrCP. (**B**) Keap1 domain structure. Asterisks in the BTB (C151) and IVR (C226, C273, and C288) domains represent cysteine residues that are targets of modification in response to oxidative stress. (**C**) p62/SQSTM1 domain structure. The asterisk in the intrinsically disordered region represents the Keap1 binding domain.

The 605-amino acid-long human Nrf2 (Figure 1A) consists of highly conserved sequences described as Nrf2-ECH homology (Neh) domains that play specific roles in the overall activity of the protein [27]. The Neh2 domain present at the amino-terminus of the protein contains two highly conserved motifs (DLG and ETGE) that interact with Keap1 [28]. The Neh1 domain located towards the carboxy-terminal region possesses a basic DNA binding domain as well as leucine zipper sequences for dimerization [29]. This region is also involved in heterodimerization with small masculo-aponeurotic fibrosarcoma (sMaf) proteins [29], which serve as transcriptional coactivators. Regions involved in interactions of Nrf2 with various factors needed in transcriptional transactivation are located in the Neh3–5 domains. While the Neh4 and Neh5 domains cooperatively recruit CREB binding protein (CBP) to genes with AREs [30], Neh3 is involved in recruiting components of the transcriptional apparatus [31]. The Neh6 domain is a target for E3 ubiquitin ligase.

β-TrCP-mediated degradation of Nrf2 [32]. Interestingly, the DNA binding domain of retinoid X receptor alpha interacts with the Neh7 and suppresses Nrf2’s transcriptional activation function [33].

### 2.2. Keap1

Keap1 is the central regulator of Nrf2 levels. It is a member of the BTB-Kelch family of proteins which assembles with the E3 ubiquitin ligase Cullin 3 (Cul3) and Rbx1 to form multiprotein complexes for protein ubiquitination. The 611-amino acid-long protein contains three major domains (Figure 1B): The BTB domain located at the amino-terminal region of the protein, the intervening region (IVR) domain in the central part of the protein, and the Kelch domain at the carboxy-terminal region [27]. While the BTB domain is involved in homodimerization, this domain along with another small region (3-box) just upstream of the IVR also interacts with Cul3 [34,35]. The homodimers of Keap1 interact through the Kelch domain with the DLG and ETGE motifs of Nrf2 [36,37,38,39].

### 2.3. p62/SQSTM1

Nrf2-Keap1 signaling is the predominant pathway employed by cells under oxidative stress conditions. However, Nrf2 activity is also regulated by other pathways such as autophagy, which is involved in maintaining cellular homeostasis by protecting cells from oxidative and proteostatic stress. The autophagy receptor p62/SQSTM1 (Figure 1C) interacts with the Kelch domain of Keap1. Since the Nrf2 binding site on Keap1 overlaps with that of the p62/SQSTM1 site, under conditions in which p62/SQSTM1 accumulates, Keap1 becomes engaged with the autophagy receptor and is degraded in the autolysosomes, thus resulting in stabilization and nuclear accumulation of Nrf2 for transcriptional activation [40]. One of the target genes of Nrf2 is the p62/SQSTM1 itself, suggesting a positive feedback loop in the activation of Nrf2 [41] by a non-canonical mechanism.

### 2.4. Mechanisms of Nrf2 Activation and Its Regulation

**Figure 2:**
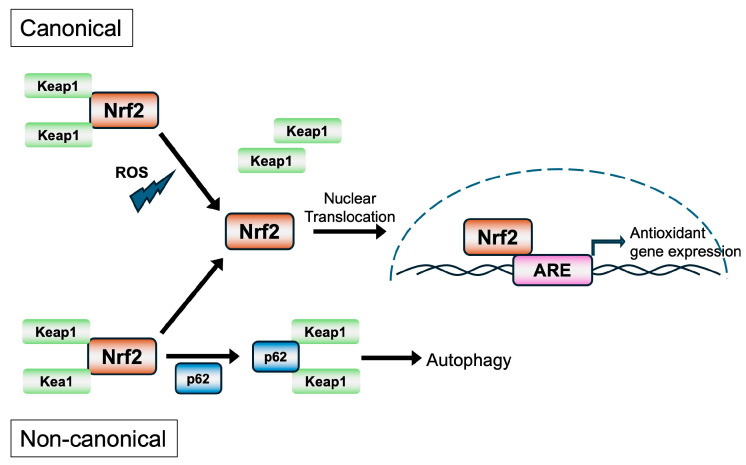
The canonical and non-canonical pathways of activation of Nrf2.

The Nrf2 signaling pathway is a central regulator of cellular redox homeostasis, responding to oxidative and electrophilic stress through transcriptional activation of cytoprotective genes. This highly conserved pathway plays a pivotal role in detoxification, antioxidant defense, inflammation modulation, metabolism, and cell survival. Activation of Nrf2 can occur predominantly through canonical mechanisms in which Keap1 dissociates from Nrf2 under oxidative or electrophilic stress, as well as non-canonical mechanisms in which Keap1 interaction with the autophagy receptor p62/SQSTM1 leads to degradation of Keap1 via autolysosomes [6] (Figure 2). Activation of Nrf2 can also occur through Keap1-independent pathways, as briefly described below.

Under basal conditions, the stable association of Nrf2 with Keap1, the cytoplasmic adaptor for the Cul3-based E3 ubiquitin ligase complex, results in constitutive ubiquitination and degradation of Nrf2 via the ubiquitin–proteasome pathway. This keeps the levels and activity of Nrf2 in check [3,42]. Keap1 functions as a redox sensor through reactive cysteine residues, particularly Cys151, Cys273, and Cys288, which are susceptible to modification by electrophiles and ROS [43,44]. In the canonical mechanism of Nrf2 activation, the “cysteine code” theory [6] proposes that upon exposure to oxidative stress or electrophilic agents, these cysteine residues become modified, leading to a conformational change in Keap1 that disrupts its ability to target Nrf2 for degradation. This results in the stabilization and accumulation of Nrf2, which translocates to the nucleus, heterodimerizes with sMaf proteins, and binds to antioxidant response elements (AREs) with core sequences of 5′-TGACNNNGC-3′ in the promoters of target genes [45,46,47]. The Nrf2-regulated genes encode a wide range of cytoprotective proteins, including phase I and II detoxification enzymes [e.g., NQO1, Glutamate–Cysteine Ligase Catalytic (GCLC) and Modifier (GCLM) subunits, GSTs], antioxidant enzymes (e.g., HO-1, SOD1, and PRDX1). These proteins facilitate the neutralization of ROS, detoxification of xenobiotics, and maintenance of glutathione and NADPH pools essential for redox balance [46,48]. Importantly, Nrf2 activity is tightly regulated not only at the post-translational level by Keap1 but also by autophagy and kinase signaling. The non-canonical mechanism does not involve modifications of the cysteine residues in Keap1 but the autophagy receptor p62/SQSTM1 interacts and sequesters Keap1 in autophagosomes, preventing Nrf2 degradation [4,40].

In Keap1-independent pathways of Nrf2 activation, kinases such as PKC, PI3K/AKT, and MAPKs modulate Nrf2 activation by phosphorylating Nrf2 or associated proteins, influencing nuclear translocation or transcriptional activation [49]. Beyond redox regulation, Nrf2 has emerging roles in metabolism, inflammation, and immunity. It modulates lipid metabolism, mitochondrial function, and autophagy, and cross-talks with inflammatory pathways such as NF-κB [50]. Persistent Nrf2 activation, while cytoprotective in acute stress, can contribute to chemoresistance, tumorigenesis, and metabolic reprogramming in cancers, highlighting its context-dependent effects [51]. Nrf2 is the master regulator of cellular defense mechanisms, orchestrating a transcriptional response that enables cells to adapt to oxidative and electrophilic stress. The Keap1–Nrf2–ARE axis represents a key signaling node with therapeutic potential in diseases characterized by oxidative stress, including neurodegenerative diseases, inflammatory conditions, and cancer.

Intricate layers of regulation of Nrf2 activity are also observed in cells that are independent of Keap1. The cytoplasmic Nrf2, free of Keap1 when phosphorylated by glycogen synthase kinase 3β (GSK-3β) at Ser335 and Ser338 residues, translocates to the nucleus, where it is ubiquitinated by β-TrCP and degraded [52]. The nuclear Nrf2 can also be phosphorylated at Tyr586 by activated Fyn kinase, which is then exported out of the nucleus and degraded in the cytoplasm [53]. Besides degradation, Nrf2 activity is also suppressed by the BTB and CNC homology 1 (Bach1) transcription factor through competition for binding sites on target DNA [54]. Down-regulation of Nrf2 activity has also been demonstrated through its deacetylation, ubiquitination, and subsequent proteosomal degradation [55] and also through promyelocytic leukemia protein (PML)-ring finger protein 4 (RNF4)-dependent pathways [56].

## 3. Nrf2 in Antiviral Immunity

In response to increased ROS production, Nrf2 is activated and leads to the establishment of an antiviral state in cells at multiple fronts by triggering antioxidant defenses, up-regulating inflammatory responses, and enhancing protective autophagy. On one hand, in cells with activated Nrf2, virus replication is compromised due to induction of an antiviral state. On the other hand, viral replication can activate Nrf2 to down-regulate oxidative stress and create a conducive environment for their replication. The role of Nrf2 in antiviral immunity has been examined for many viruses but here we will elaborate on studies with few selected important RNA and DNA viruses (Table 1).

Studies on the influenza A virus (IAV) have shown an increase in the production of ROS, activation of Nrf2 pathway and its downstream effector HO-1 coupled with a decrease in intracellular glutathione (GSH) [1,57,58]. The antioxidant response mediated by Nrf2 protects the lungs of mice against oxidative damage and inflammation [59]. A key player in the Nrf2-mediated antioxidant response is HO-1, an anti-inflammatory enzyme. In response to increased production of oxidants in the cell, HO-1 is induced, leading to heme degradation and production of free iron, (Fe^2+^), carbon monoxide, and biliverdin. HO-1 has been shown to increase infiltration of macrophages and up-regulate type I interferon signaling, thereby producing an antiviral environment in lung tissue infected with IAV [60]. Sulforaphen (SFN), emodin, or itaconate derivatives, which induce or activate Nrf2, or compounds such as bakuchiol and YZH-106, which activate Nrf2, have been shown to inhibit oxidative stress and suppress IAV replication [61,62,63,64]. Additionally, it was shown that overexpression of Nrf2 can also inhibit IAV entry into cells [65]. IAV also down-regulates Nrf2 by decreasing the expression and activity of glucose-6-phosphate dehydrogenase (G6PD) [66]. Overall, these studies, along with other studies reported in the literature, demonstrate that activation of the Nrf2 signaling pathway is critical for suppressing IAV replication and pathogenesis. The suppression of IAV replication and virus-induced pneumonia has been shown at the level of virus adsorption and entry [64,65] and in nuclear export of viral components [62,63], or by activating Nrf2 pathway and inhibiting activation of the NF-kB, TLR4, and p38/JNK MAPK pathways [61].

Dengue virus (DENV) has been shown to induce production of ROS and oxidative stress, resulting in the release of inflammatory cytokines, which is a major contributor to disease pathogenesis [67,68,69]. In response to increased ROS, the Nrf2 pathway is activated to counter the oxidative stress conditions. Nrf2 effector antioxidant enzymes like HO-1, GCLC, superoxide dismutase 2 (SOD2), and others are up-regulated [70,71]. Furthermore, it was demonstrated that Nrf2 activation in DENV-infected cells is achieved by the activation of protein kinase R-like ER kinase (PERK), a kinase associated with ER stress [71]. With Zika virus, a close relative of DENV, induction of HO-1 and establishment of innate host defense due to Nrf2 was shown to inhibit ZIKV replication [72]. In a more recent study, significantly higher levels of replication were observed in ZIKV-infected cells depleted of Nrf2, demonstrating that Nrf2 and its signaling pathways are antagonistic to ZIKV replication [10].

RSV is known to induce oxidative stress in infected cells and activate Nrf2 and its downstream effector HO-1, which inhibit virus replication [73,74,75,76]. Cobalt protoporphyrin (CoPP) is a known activator of Nrf2 and has been shown to induce expression of HO-1 [77]. Studies in mice have shown that induction of HO-1 by CoPP up-regulated IFN-α/β and reduced RSV load in lung tissue [75]. Other studies have demonstrated that the activation of Nrf2 in RSV-infected cells occurs early in the infection cycle [55,78] while in later stages of infection, Nrf2 experiences degradation by Keap1-dependent [55] and-independent pathways [56]. Using an agonist [butylated hydroxyanisole (BHA)] and an inhibitor [trigonelline (TRI)] of the Nrf2-ARE pathway, up-regulation of TLR7, mediated by the Nrf2-ARE pathway, was observed in RSV-infected cells [78]. The study revealed that activation of Nrf2 results in down-regulation of RSV replication in cells in vitro [78]. Furthermore, the antiviral activity of Nrf2 was demonstrated in a murine model of respiratory syncytial virus disease [79].

With the emergence of severe acute respiratory syndrome coronavirus 2 (SARS-CoV-2) and the COVID pandemic, infection with the virus was shown to induce high levels of oxidative stress. SARS-CoV-2 infection was found to significantly down-regulate Nrf2 expression and antioxidant responses in many different cell types in vitro and in the lungs of a murine model of infection [80]. Conversely, activators of Nrf2 such as 4-octyl-itaconate (4-OI), bardoxolone, and SFN have been shown to restrict SARS-CoV-2 infection. While these compounds are known Nrf2 activators, the mechanism of virus inhibition can be independent of Nrf2 [81]. Bardoxolone and methyl bardoxolone were demonstrated to significantly inhibit replication of SARS-CoV-2 [82]. Another clinically approved Nrf2 agonist, DMF, has also been shown to inhibit SARS-Cov-2 replication in different cell lines by suppressing the release of pro-inflammatory cytokines [83]. Overall, these studies suggest that Nrf2 functions in protecting cells from viral infection and point to an antiviral role for Nrf2 in SARS-CoV-2 infection. To counter the antiviral effects, the ORF3a and ORF6 proteins of SARS-CoV-2 have been shown to reduce the levels of Nrf2 and interfere with the cellular redox homeostasis [84,85].

Nrf2 has also been shown to have antiviral effects on replication of human immunodeficiency virus type 1 (HIV-1). Oxidative stress is induced in response to HIV infection and results in redox imbalance, a key determinant of HIV-induced pathogenicity [86,87,88,89]. Several HIV-1 proteins such as tat, nef, vpr, and gp120 have been shown to induce oxidative stress [89]. HIV-1 tat protein was shown to induce Nrf2 activation in neuronal cells [90] but, in a separate study, both tat and gp120 were found to suppress Nrf2 in human monocyte-derived macrophages [91]. Nrf2 activator methyl bardoxolone was found to inhibit HIV-1 replication in cells [92]. Overall, these studies point towards an antiviral role for Nrf2 against HIV.

Chronic infection with HCV is known to induce oxidative stress in human liver cells. In these cells, HCV proteins activate Nrf2 through phosphorylation by PKC, MAPK, and PI3K and subsequent nuclear translocation [93]. The role of Nrf2 in HCV replication appears to be both antiviral as well as proviral. Since HCV replication is closely tied to lipid metabolism and formation of lipid droplets, activated Nrf2 seems to play a critical proviral role in HCV replication. Silencing Nrf2 significantly reduced HCV replication in persistently infected cell lines [94]. However, it was also observed that inhibition of Nrf2 triggered autophagy and favored HCV particle release [95,96]. In cells supporting HCV replication, Nrf2 levels in the nucleus and Nrf2/ARE-dependent gene expression were significantly reduced [97], suggesting a role for Nrf2 in limiting infectious progeny production.

Studies with Hepatitis B virus (HBV), a DNA virus, have shown induction of oxidative stress and Nrf2 activation upon infection [9,98,99]. HBV proteins HBx and LHBs induce expression of Nrf2 [98], thereby leading to inhibition of HBV replication. It has been suggested that p62/Keap1 interaction in HBV-infected cells leads to activation of Nrf2 mediated by HBx [100]. A recent study demonstrated that use of Nrf2 activators results in suppression of HBV replication, and this is achieved by Nrf2-mediated induction of HO-1 [99]. These studies point to an antiviral effect of Nrf2 in HBV infection.

Recent studies with herpes simplex virus 1 (HSV-1) have demonstrated that upon infection, HSV-1 triggers an Nrf2-mediated antiviral response in cells resulting in inhibition of virus replication [101,102,103]. Wyler et al. [101] used single-cell RNA sequencing to study host cell genes and pathways that are impacted in HSV-1-infected cells and found that the Nrf2 pathway correlates with resistance to infection. By treating cells with Nrf2 agonists, they were able to demonstrate that up-regulation of Nrf2 leads to inhibition of HSV-1 replication [101]. Another study has shown that, in early phases of HSV-1 infection, accumulation of ROS and activation of Nrf2 and its downstream effectors HO-1 and NQO1 occur. Treatment of cells with Nrf2 agonists like tert-butylhydroquinone (tBHQ) or ginsenoside Rg5 led to an inhibitory effect on virus replication [102,103]. Additionally, ginsenoside Rg5 had a cytoprotective effect on cells infected with HSV-1 by suppressing inflammatory cytokine production [103]. Nrf2 knockout mice were found to be more susceptible to murine cytomegalovirus (MCMV) compared to wild-type mice, further establishing that Nrf2 functions as an antiviral host protein against MCMV [104]. However, studies with human CMV suggest that activation of Nrf2 and consequent increase in HO-1 appear to be beneficial for virus replication by enhancing the host cell’s ability to handle oxidative stress induced by virus replication [105].

It appears that in the vast majority of viral infections, Nrf2 acts as an antiviral molecule to limit virus replication and protect infected cells and animals from virus-induced pathogenesis. The mechanisms that viruses adopt to induce Nrf2 vary widely, as noted above.

## 4. Nrf2 in Proviral Roles

The activation of Nrf2 in the context of virus infection has been implicated in the establishment of an antiviral state in host cells, but studies have also demonstrated that Nrf2 activation can lead to a cellular environment that is conducive to virus replication. While evidence of Nrf2 directly serving a proviral role is lacking, viruses have evolved mechanisms that exploit the effects of Nrf2 activation to their advantage (Table 1). The Nrf2 agonist, 4-OI, is known to reduce inflammation and down-regulate type I IFN responses [106]. It has also been shown to engage Nrf2 and suppress STING activation [107]. Such an environment can be exploited by viruses for efficient viral replication. Indeed, a recent oncolytic virotherapy study with VSVΔ51 demonstrated that when cells are treated with 4-OI, there is a significant increase in VSV replication [108]. Studies on HIV have demonstrated that the viral reverse transcriptase up-regulates HO-1 and NQO1 and suppresses the expression of IFN-γ and IL-2. While the host immune response is dampened, cellular redox balance is restored, which contributes to cell survival and HIV persistence [73,109]. The ORF8-encoded protein of SARS-CoV-2 activates the unfolded protein response (UPR) pathways [110]. It has been reported that activation of UPR results in PERK-mediated phosphorylation and nuclear translocation of Nrf2, thereby increasing cell survival [111]. Together, these events induce a proviral state in cells that is exploited by many respiratory viruses including coronaviruses, RSV, rhinoviruses, and influenza viruses for their replication and pathogenesis [73]. A study using hepatocellular carcinoma cells positive for HCV found that HCV-mediated Nrf2 induction can lead to changes in the metabolism of the cells that promote virus replication, tumor growth, and drug resistance [112].

In the case of HBV, the viral protein HBx, through the Nrf2 pathway, has been shown to interact with two subunits of the 26S immunoproteasome and reduce its activity [73,113]. This results in inhibition of antigen processing and presentation, allowing the virus to escape host immunity. ASFV induces oxidative stress that serves a proviral role. Upon ASFV infection, accumulation of ROS leads to Nrf2-mediated activation of GCLC, a downstream effector of Nrf2 involved in glutathione synthesis and regeneration of reduced glutathione in cells [23]. An overall increase in the cellular GSH content results in neutralization of ROS, thereby maintaining an environment that facilitates ASFV replication [23]. In cells infected with vaccinia virus, Nrf2 is activated, leading to expression of antioxidant genes that balance the redox state of the infected cell [24]. This virus-induced balance has been suggested as serving an immunomodulatory mechanism that dampens the host defense and enhances virus replication [24]. The Nrf2 pathway has also been implicated to have a proviral role in KSHV infection and Kaposi’s sarcoma (KS) progression. Activation of Nrf2 in KSHV-infected cells creates an environment conducive to virus replication [26]. A recent study revealed that KSHV infection leads to Nrf2-mediated HO-1 induction and that HO-1 is highly expressed in KS lesions [114]. Additionally, the study also demonstrates that viral G protein-coupled receptors (vGPCRs) are also involved in activation and nuclear translocation of Nrf2 [114]. These findings provide evidence that Nrf2 plays a proviral role not only in virus infection but also in cancer progression.

**Table 1:** Pro- and antiviral roles of Nrf2 in RNA and DNA viruses.

RNA Viruses				
Virus	Nrf2 Status	Key Nrf2-Related Mechanisms	Antiviral or Proviral	Citations
IAV	Activation	Antioxidant response reduces oxidative stress; HO-1 increases macrophage infiltration and IFN-I; Nrf2 activation inhibits viral entry, nuclear export, and pro-inflammatory pathways (NF-κB, MAPK, TLR4)	Antiviral	[1,57,58,59,60,61,62,63,64,65,66]
DENV	Activation	Nrf2 activation counteracts oxidative stress and reduces inflammatory damage	Antiviral	[67,68,69,70,71]
ZIKV	Activation	Nrf2 depletion increases viral replication; HO-1 contributes to antiviral state	Antiviral	[10,72]
RSV	Early activation; later degradation	Nrf2 activation reduces RSV replication; HO-1 induction stimulates IFN-α/β and reduces lung viral load; Nrf2 agonists suppress replication	Antiviral	[55,56,73,74,75,76,77,78,79]
SARS-CoV-2	Suppression	Viral proteins ORF3a/ORF6 inhibit Nrf2; Nrf2 activators (4-OI, DMF, bardoxolone) strongly inhibit replication	Antiviral	[80,84,85,115]
HIV-1	Both (context-dependent)	Nrf2 activation inhibits HIV replication (bardoxolone); HIV proteins induce oxidative stress	Antiviral	[59,86,87,88,89,90,91,92]
HCV	Activation	Nrf2 supports lipid metabolism needed for replication (proviral); but also limits production of infectious progeny; Nrf2 inhibition promotes autophagy and virion release	Both (context-dependent)	[93,94,95,96,97]
**DNA Viruses**				
HBV	Activation	Nrf2 activation suppresses HBV replication; HO-1 induction is antiviral	Antiviral	[9,73,98,99,100,113]
HSV-1	Activation	Nrf2 activation limits replication; Nrf2 agonists (tBHQ, Rg5) strongly inhibit HSV-1	Antiviral	[101,102,103]
MCMV	Activation	Nrf2 knockout mice highly susceptible	Antiviral	[104]
HCMV	Activation	Nrf2/HO-1 induction enhances host tolerance to oxidative stress, which supports viral replication	Proviral	[105]
ASFV	Activation	Increased GSH neutralizes ROS and creates environment favorable for ASFV replication	Proviral	[23]
Vaccinia Virus	Activation	Nrf2-driven antioxidant gene expression dampens host defense; creates redox-balanced environment beneficial for replication	Proviral	[24]
KSHV	Activation	Nrf2 activation and HO-1 induction promote viral replication and KS tumor progression	Proviral	[26,114]
VSVΔ51 (Oncolytic VSV)	Activation	Enhanced viral replication	Proviral	[106,107,108]

## 5. Therapeutic Targeting of Nrf2 in Viral Infections

Virus infection and replication in cells are known to be associated with induction of inflammation as well as oxidative and nitrosative stress [1,73]. Since the Nrf2 signaling pathway is a major contributor to maintaining the redox state of a cell, targeting Nrf2 by therapeutics has been a promising strategy in treating viral infections. As described in the sections above, Nrf2 serves an antiviral role in a large number of viral infections, although for some viruses, it plays a proviral role. Therefore, therapeutics that activate Nrf2 have become the logical choice for use in limiting virus infections. Since Nrf2 can be activated by several different mechanisms (Table 2), the therapeutics and drugs can be classified into three major classes based on their mode of action. The class of Nrf2 activators (or agonists) that covalently modify the cysteine residues (particularly, C151, C273, and C288) in Keap1 protein are referred to as “electrophilic compounds” [115,116]. Another class of activators includes those that compromise protein–protein interactions (PPI) between Nrf2 and Keap1 or between Keap1 and p62/SQSTM1. A third class of Nrf2 activators inhibits cellular proteins that phosphorylate and degrade Nrf2 such as GSK-3 and β-TrCP [115]. However, the predominant class of Nrf2 activators that has been demonstrated to have antiviral properties is the class of electrophilic compounds.

Studies have shown that Nrf2 agonists such as 4-OI and DMF were able to reduce inflammation caused by SARS-CoV-2 infection [73,83]. Interaction of 4-OI with Keap1 prevents Keap1 from binding to Nrf2, resulting in Nrf2-mediated activation of antioxidant genes such as HO-1 and GSH [106]. It is also known to inhibit STING and IFN signaling [107]. DMF has been FDA-approved for use in the treatment of multiple sclerosis but has been shown to be effective against SARS-CoV-2-induced inflammation as well [83]. Additionally, BHA and tBHQ have been shown to activate Nrf2 and associated genes, resulting in reduced RSV [55] and HSV-1 [102] replication. The isothiocyanate SFN, a naturally occurring Nrf2 inducer found in broccoli, inhibits IAV infection and replication [73,117,118] and RSV replication [79]. Carbocisteine [119] and epigallocatechin-3-0-galate (EGCG) [120] are other Nrf2 inducers that have shown anti-influenza activity. Furthermore, curcumin is also known to activate Nrf2 and its downstream effector HO-1, leading to reduced replication of IAV [121], parainfluenza [122], and RSV [123]. Another natural compound, Bakuchiol, is also able to activate Nrf2 and inhibit IAV by reducing viral mRNA and protein synthesis [124,125]. Nrf2 agonists may also inhibit IAV replication by directly inhibiting the translocation of IAV ribonucleoprotein (vRNP) from the nucleus to the cytoplasm. This process is independent of the Nrf2 signaling pathway [62].

Among the Nrf2 activators that compromise PPI are small peptides derived from regions of Nrf2 that interact with Keap1 [126] or hybrid peptides derived based on sequences spanning the Keap1 interaction region on Nrf2 (ETGE motif) and region of interaction between Keap1 and p62/SQSTM1 [127]. In addition to these peptide inhibitors of PPI, several small-molecule activators of Nrf2, acting at the level of Keap1-Nrf2 PPI such as tetrahydroisoquinoline [128], naphthalene, and others [128,129,130,131], have also been developed. However, their use in inhibiting virus infections is limited. In one study, naphthalene derivatives were found to possess antiviral properties against IAV [132], but their mechanism of action remains unknown.

## 6. Outstanding Questions and Future Directions

Despite major advances in understanding the interplay between Nrf2 signaling and viral infections, disease pathogenesis, and cancer progression, critical gaps remain, needing further exploration. One priority is the development of virus- and tissue-specific modulators of Nrf2. Current pharmacological activators, such as SFN or DMF, broadly up-regulate Nrf2 across diverse tissues, which may be beneficial against certain viruses but deleterious in others or in the context of cancer. Future drug discovery efforts should be aimed at designing context-specific drugs that can either activate or inhibit Nrf2 depending on the viral pathogen and the cell type. Another pressing area involves clarifying Nrf2’s role in immunometabolism and viral latency and reactivation. Nrf2 regulates not only antioxidant pathways but also glycolysis, glutaminolysis, and lipid metabolism, all of which are critical for both innate immune responses and viral replication. Yet, a lack of comprehensive studies on how Nrf2-driven metabolic rewiring affects antiviral defense versus proviral support across different viral families still exists. Investigating whether Nrf2 activation favors viral persistence or immune control could lead to the uncovering of novel strategies to prevent reactivation-related disease. Additionally, there is a strong need to expand clinical trials with drug candidates evaluating Nrf2 modulators in infectious disease settings. Small-scale studies in influenza and SARS-CoV-2 have provided preliminary evidence that Nrf2 activators can reduce viral burden and inflammation, but larger, well-controlled clinical trials are needed to establish efficacy, safety, and optimal dosing regimens. A major bottleneck is the lack of virus-specific mechanistic studies. Nrf2 is broadly labeled as “antiviral” or “proviral” without a detailed understanding of the exact viral proteins, host cofactors, or signaling cross-talk involved. Detailed mechanistic analyses are essential to pinpoint how different viruses exploit the Nrf2 pathway. Finally, a long-term challenge is to integrate Nrf2 biology into a co-evolution of host and pathogen. Viruses evolve under selective pressure to manipulate redox signaling, while hosts adapt by refining Nrf2 pathways to balance protection against oxidative stress with control of infection. Together, these outstanding questions highlight the need for interdisciplinary approaches spanning redox biology, virology, immunology, and clinical medicine to fully understand Nrf2 as both a therapeutic target and a window into virus–host co-evolution.

## 7. Conclusions

Nrf2 has emerged as a central regulator linking redox balance, host defense, and viral pathogenesis. Viral infections often elevate ROS, which can damage host cells but may also facilitate replication. Nrf2 counters this stress by up-regulating antioxidant and cytoprotective genes such as HO-1, NQO1, and those driving glutathione metabolism, thereby shaping infection outcomes. Recent studies highlight the dual roles of Nrf2 as an antiviral in many settings but a proviral in others. In RNA virus infections, Nrf2 generally exerts antiviral effects. Activation by pharmacologic agents including SFN, DMF, or itaconate derivatives suppresses replication of influenza A virus, respiratory syncytial virus, and SARS-CoV-2 by reducing oxidative stress and inflammation. In contrast, vesicular stomatitis virus replicates efficiently in Nrf2-high tumor environments, where the antioxidant state supports oncolysis. In the case of ASFV and vaccinia virus, Nrf2 is activated to enhance antioxidant capacity and replication. Beyond infection, Nrf2 hyperactivation is a hallmark of cancer, conferring survival and therapy resistance. Oncogenic viruses such as KSHV engage Nrf2 to sustain transformation, while oncolytic viruses exploit Nrf2-high states for replication. Nrf2 activators hold promise as host-directed antivirals, but their context-dependent effects highlight therapeutic complexity. In some cases, activation may favor viral persistence or tumor growth, while inhibition risks exacerbating oxidative injury. Thus, Nrf2 represents a true double-edged sword, demanding virus- and tissue-specific strategies to harness its protective functions without promoting viral fitness or cancer progression. Understanding the nuances of Nrf2 signaling in viral infections will enable more precise, personalized approaches to antiviral therapy that harness its protective functions while avoiding its exploitation by viruses.

## Figures and Tables

**Table 2:** Classes of Nrf2 activators and their mechanisms.

Activator Class	Primary Mechanism	Example Compounds
Electrophilic KEAP1 modifiers	Cysteine modification → KEAP1 inactivation	Sulforaphane, DMF, 4-OI, BHA, tBHQ, Carbocisteine
KEAP1–Nrf2 PPI inhibitors	Block Kelch domain binding	PRL-295, KI-696
GSK-3β inhibitors	Block β-TrCP degradation	Lithium Chloride, CHIR99021
Natural products	Mixed electrophilic/kinase actions	EGCG, curcumin, resveratrol

## Data Availability

No new data was generated from this research.

## References

[1] Kayesh M.E.H., Kohara M., Tsukiyama-Kohara K. (2025). Effects of oxidative stress on viral infections: An overview. npj Viruses.

[2] Krishnamurthy H.K., Pereira M., Rajavelu I., Jayaraman V., Krishna K., Wang T., Bei K., Rajasekaran J.J. (2024). Oxidative stress: Fundamentals and advances in quantification techniques. Front. Chem..

[3] McCord J.M., Gao B., Hybertson B.M. (2023). The Complex Genetic and Epigenetic Regulation of the Nrf2 Pathways: A Review. Antioxidants.

[4] Komatsu M., Kurokawa H., Waguri S., Taguchi K., Kobayashi A., Ichimura Y., Sou Y.-S., Ueno I., Sakamoto A., Tong K.I. (2010). The selective autophagy substrate p62 activates the stress responsive transcription factor Nrf2 through inactivation of Keap1. Nat. Cell Biol..

[5] Yasuda M., Ohzeki Y., Shimizu S., Naito S., Ohtsuru A., Yamamoto T., Kuroiwa Y. (1999). Stimulation of in vitro angiogenesis by hydrogen peroxide and the relation with ETS-1 in endothelial cells. Life Sci..

[6] Zhang D.D. (2025). Thirty years of NRF2: Advances and therapeutic challenges. Nat. Rev. Drug Discov..

[7] Hayes J.D., Dinkova-Kostova A.T. (2014). The Nrf2 regulatory network provides an interface between redox and intermediary metabolism. Trends Biochem. Sci..

[8] Pi X., Xie L., Portbury A.L., Kumar S., Lockyer P., Li X., Patterson C. (2014). NADPH oxidase-generated reactive oxygen species are required for stromal cell-derived factor-1alpha-stimulated angiogenesis. Arter. Thromb. Vasc. Biol..

[9] Herengt A., Thyrsted J., Holm C.K. (2021). NRF2 in Viral Infection. Antioxidants.

[10] Sahoo B.R., Crook A.A., Pattnaik A., Torres-Gerena A.D., Khalimonchuk O., Powers R., Franco R., Pattnaik A.K. (2023). Redox Regulation and Metabolic Dependency of Zika Virus Replication: Inhibition by Nrf2-Antioxidant Response and NAD(H) Antimetabolites. J. Virol..

[11] Ramos-Gomez M., Kwak M.K., Dolan P.M., Itoh K., Yamamoto M., Talalay P., Kensler T.W. (2001). Sensitivity to carcinogenesis is increased and chemoprotective efficacy of enzyme inducers is lost in nrf2 transcription factor-deficient mice. Proc. Natl. Acad. Sci. USA.

[12] Kwak M.K., Itoh K., Yamamoto M., Sutter T.R., Kensler T.W. (2001). Role of transcription factor Nrf2 in the induction of hepatic phase 2 and antioxidative enzymes in vivo by the cancer chemoprotective agent, 3H-1, 2-dimethiole-3-thione. Mol. Med..

[13] Mitsuishi Y., Taguchi K., Kawatani Y., Shibata T., Nukiwa T., Aburatani H., Yamamoto M., Motohashi H. (2012). Nrf2 redirects glucose and glutamine into anabolic pathways in metabolic reprogramming. Cancer Cell.

[14] Vomund S., Schafer A., Parnham M.J., Brune B., von Knethen A. (2017). Nrf2, the Master Regulator of Anti-Oxidative Responses. Int. J. Mol. Sci..

[15] Anandhan A., Dodson M., Shakya A., Chen J., Liu P., Wei Y., Tan H., Wang Q., Jiang Z., Yang K. (2023). NRF2 controls iron homeostasis and ferroptosis through HERC2 and VAMP8. Sci. Adv..

[16] Moi P., Chan K., Asunis I., Cao A., Kan Y.W. (1994). Isolation of NF-E2-related factor 2 (Nrf2), a NF-E2-like basic leucine zipper transcriptional activator that binds to the tandem NF-E2/AP1 repeat of the beta-globin locus control region. Proc. Natl. Acad. Sci. USA.

[17] Itoh K., Igarashi K., Hayashi N., Nishizawa M., Yamamoto M. (1995). Cloning and characterization of a novel erythroid cell-derived CNC family transcription factor heterodimerizing with the small Maf family proteins. Mol. Cell Biol..

[18] McMahon M., Itoh K., Yamamoto M., Hayes J.D. (2003). Keap1-dependent proteasomal degradation of transcription factor Nrf2 contributes to the negative regulation of antioxidant response element-driven gene expression. J. Biol. Chem..

[19] Kobayashi A., Kang M.I., Okawa H., Ohtsuji M., Zenke Y., Chiba T., Igarashi K., Yamamoto M. (2004). Oxidative stress sensor Keap1 functions as an adaptor for Cul3-based E3 ligase to regulate proteasomal degradation of Nrf2. Mol. Cell Biol..

[20] Cullinan S.B., Gordan J.D., Jin J., Harper J.W., Diehl J.A. (2004). The Keap1-BTB protein is an adaptor that bridges Nrf2 to a Cul3-based E3 ligase: Oxidative stress sensing by a Cul3-Keap1 ligase. Mol. Cell Biol..

[21] Itoh K., Wakabayashi N., Katoh Y., Ishii T., O’Connor T., Yamamoto M. (2003). Keap1 regulates both cytoplasmic-nuclear shuttling and degradation of Nrf2 in response to electrophiles. Genes Cells.

[22] Furukawa M., Xiong Y. (2005). BTB protein Keap1 targets antioxidant transcription factor Nrf2 for ubiquitination by the Cullin 3-Roc1 ligase. Mol. Cell Biol..

[23] Gao H., Gu T., Gao X., Song Z., Liu J., Song Y., Zhang G., Sun Y. (2024). African swine fever virus enhances viral replication by increasing intracellular reduced glutathione levels, which suppresses stress granule formation. Vet. Res..

[24] da Silva Menegatto M.B., Ferraz A.C., Lima R.L.S., Guimaraes P.H., Ola-Olu O.S., Machado-Junior P.A., Carvalho Malta W., de Fatima Silva Moraes T., Silva Bezerra F., de Mello Silva B. (2025). Vaccinia virus modulates the redox environment by inhibiting reactive oxygen and nitrogen species with increased activity of endogenous antioxidant enzymes. Sci. Rep..

[25] Gjyshi O., Flaherty S., Veettil M.V., Johnson K.E., Chandran B., Bottero V. (2015). Kaposi’s sarcoma-associated herpesvirus induces Nrf2 activation in latently infected endothelial cells through SQSTM1 phosphorylation and interaction with polyubiquitinated Keap1. J. Virol..

[26] Gjyshi O., Bottero V., Veettil M.V., Dutta S., Singh V.V., Chikoti L., Chandran B. (2014). Kaposi’s sarcoma-associated herpesvirus induces Nrf2 during de novo infection of endothelial cells to create a microenvironment conducive to infection. PLoS Pathog..

[27] Canning P., Sorrell F.J., Bullock A.N. (2015). Structural basis of Keap1 interactions with Nrf2. Free Radic. Biol. Med..

[28] Tong K.I., Katoh Y., Kusunoki H., Itoh K., Tanaka T., Yamamoto M. (2006). Keap1 recruits Neh2 through binding to ETGE and DLG motifs: Characterization of the two-site molecular recognition model. Mol. Cell Biol..

[29] Motohashi H., Katsuoka F., Engel J.D., Yamamoto M. (2004). Small Maf proteins serve as transcriptional cofactors for keratinocyte differentiation in the Keap1-Nrf2 regulatory pathway. Proc. Natl. Acad. Sci. USA.

[30] Katoh Y., Itoh K., Yoshida E., Miyagishi M., Fukamizu A., Yamamoto M. (2001). Two domains of Nrf2 cooperatively bind CBP, a CREB binding protein, and synergistically activate transcription. Genes Cells.

[31] Nioi P., Nguyen T., Sherratt P.J., Pickett C.B. (2005). The carboxy-terminal Neh3 domain of Nrf2 is required for transcriptional activation. Mol. Cell Biol..

[32] McMahon M., Thomas N., Itoh K., Yamamoto M., Hayes J.D. (2004). Redox-regulated turnover of Nrf2 is determined by at least two separate protein domains, the redox-sensitive Neh2 degron and the redox-insensitive Neh6 degron. J. Biol. Chem..

[33] Wang H., Liu K., Geng M., Gao P., Wu X., Hai Y., Li Y., Li Y., Luo L., Hayes J.D. (2013). RXRalpha inhibits the NRF2-ARE signaling pathway through a direct interaction with the Neh7 domain of NRF2. Cancer Res..

[34] Cleasby A., Yon J., Day P.J., Richardson C., Tickle I.J., Williams P.A., Callahan J.F., Carr R., Concha N., Kerns J.K. (2014). Structure of the BTB domain of Keap1 and its interaction with the triterpenoid antagonist CDDO. PLoS ONE.

[35] Canning P., Cooper C.D.O., Krojer T., Murray J.W., Pike A.C.W., Chaikuad A., Keates T., Thangaratnarajah C., Hojzan V., Marsden B.D. (2013). Structural basis for Cul3 protein assembly with the BTB-Kelch family of E3 ubiquitin ligases. J. Biol. Chem..

[36] Lo S.C., Li X., Henzl M.T., Beamer L.J., Hannink M. (2006). Structure of the Keap1:Nrf2 interface provides mechanistic insight into Nrf2 signaling. EMBO J..

[37] Padmanabhan B., Tong K.I., Ohta T., Nakamura Y., Scharlock M., Ohtsuji M., Kang M.I., Kobayashi A., Yokoyama S., Yamamoto M. (2006). Structural basis for defects of Keap1 activity provoked by its point mutations in lung cancer. Mol. Cell.

[38] Tong K.I., Padmanabhan B., Kobayashi A., Shang C., Hirotsu Y., Yokoyama S., Yamamoto M. (2007). Different electrostatic potentials define ETGE and DLG motifs as hinge and latch in oxidative stress response. Mol. Cell Biol..

[39] Fukutomi T., Takagi K., Mizushima T., Ohuchi N., Yamamoto M. (2014). Kinetic, thermodynamic, and structural characterizations of the association between Nrf2-DLGex degron and Keap1. Mol. Cell Biol..

[40] Lau A., Wang X.J., Zhao F., Villeneuve N.F., Wu T., Jiang T., Sun Z., White E., Zhang D.D. (2010). A noncanonical mechanism of Nrf2 activation by autophagy deficiency: Direct interaction between Keap1 and p62. Mol. Cell Biol..

[41] Katsuragi Y., Ichimura Y., Komatsu M. (2016). Regulation of the Keap1–Nrf2 pathway by p62/SQSTM1. Curr. Opin. Toxicol..

[42] Baird L., Yamamoto M. (2020). The Molecular Mechanisms Regulating the KEAP1-NRF2 Pathway. Mol. Cell Biol..

[43] Zhang D.D., Hannink M. (2003). Distinct cysteine residues in Keap1 are required for Keap1-dependent ubiquitination of Nrf2 and for stabilization of Nrf2 by chemopreventive agents and oxidative stress. Mol. Cell Biol..

[44] Gatbonton-Schwager T., Yagishita Y., Joshi T., Wakabayashi N., Srinivasan H., Suzuki T., Yamamoto M., Kensler T.W. (2023). A Point Mutation at C151 of Keap1 of Mice Abrogates NRF2 Signaling, Cytoprotection In Vitro, and Hepatoprotection In Vivo by Bardoxolone Methyl (CDDO-Me). Mol. Pharmacol..

[45] Yamamoto M., Kensler T.W., Motohashi H. (2018). The KEAP1-NRF2 System: A Thiol-Based Sensor-Effector Apparatus for Maintaining Redox Homeostasis. Physiol. Rev..

[46] Morgenstern C., Lastres-Becker I., Demirdogen B.C., Costa V.M., Daiber A., Foresti R., Motterlini R., Kalyoncu S., Arioz B.I., Genc S. (2024). Biomarkers of NRF2 signalling: Current status and future challenges. Redox Biol..

[47] Rushmore T.H., Morton M.R., Pickett C.B. (1991). The antioxidant responsive element. Activation by oxidative stress and identification of the DNA consensus sequence required for functional activity. J. Biol. Chem..

[48] Tonelli C., Chio I.I.C., Tuveson D.A. (2018). Transcriptional Regulation by Nrf2. Antioxid. Redox Signal..

[49] de la Vega M.R., Chapman E., Zhang D.D. (2018). NRF2 and the Hallmarks of Cancer. Cancer Cell.

[50] Muzammil K., Sabah Ghnim Z., Saeed Gataa I., Fawzi Al-Hussainy A., Ali Soud N., Adil M., Ali Shallan M., Yasamineh S. (2024). NRF2-mediated regulation of lipid pathways in viral infection. Mol. Asp. Med..

[51] Cuadrado A., Rojo A.I., Wells G., Hayes J.D., Cousin S.P., Rumsey W.L., Attucks O.C., Franklin S., Levonen A.L., Kensler T.W. (2019). Therapeutic targeting of the NRF2 and KEAP1 partnership in chronic diseases. Nat. Rev. Drug Discov..

[52] Hayes J.D., Chowdhry S., Dinkova-Kostova A.T., Sutherland C. (2015). Dual regulation of transcription factor Nrf2 by Keap1 and by the combined actions of beta-TrCP and GSK-3. Biochem. Soc. Trans..

[53] Dai X., Yan X., Zeng J., Chen J., Wang Y., Chen J., Li Y., Barati M.T., Wintergerst K.A., Pan K. (2017). Elevating CXCR7 Improves Angiogenic Function of EPCs via Akt/GSK-3beta/Fyn-Mediated Nrf2 Activation in Diabetic Limb Ischemia. Circ. Res..

[54] Kaspar J.W., Jaiswal A.K. (2010). Antioxidant-induced phosphorylation of tyrosine 486 leads to rapid nuclear export of Bach1 that allows Nrf2 to bind to the antioxidant response element and activate defensive gene expression. J. Biol. Chem..

[55] Komaravelli N., Tian B., Ivanciuc T., Mautemps N., Brasier A.R., Garofalo R.P., Casola A. (2015). Respiratory syncytial virus infection down-regulates antioxidant enzyme expression by triggering deacetylation-proteasomal degradation of Nrf2. Free Radic. Biol. Med..

[56] Komaravelli N., Ansar M., Garofalo R.P., Casola A. (2017). Respiratory syncytial virus induces NRF2 degradation through a promyelocytic leukemia protein—Ring finger protein 4 dependent pathway. Free Radic. Biol. Med..

[57] Lin X., Wang R., Zou W., Sun X., Liu X., Zhao L., Wang S., Jin M. (2016). The Influenza Virus H5N1 Infection Can Induce ROS Production for Viral Replication and Host Cell Death in A549 Cells Modulated by Human Cu/Zn Superoxide Dismutase (SOD1) Overexpression. Viruses.

[58] Kosmider B., Messier E.M., Janssen W.J., Nahreini P., Wang J., Hartshorn K.L., Mason R.J. (2012). Nrf2 protects human alveolar epithelial cells against injury induced by influenza A virus. Respir. Res..

[59] Yageta Y., Ishii Y., Morishima Y., Masuko H., Ano S., Yamadori T., Itoh K., Takeuchi K., Yamamoto M., Hizawa N. (2011). Role of Nrf2 in host defense against influenza virus in cigarette smoke-exposed mice. J. Virol..

[60] Ma L., Zhang P., Li X., Sun B., Li Y., Jiang J. (2025). Dual role of HO-1 in mediating antiviral immune responses and mitigating excessive inflammatory damage during influenza virus infection. iScience.

[61] Dai J.P., Wang Q.W., Su Y., Gu L.M., Zhao Y., Chen X.X., Chen C., Li W.Z., Wang G.F., Li K.S. (2017). Emodin Inhibition of Influenza A Virus Replication and Influenza Viral Pneumonia via the Nrf2, TLR4, p38/JNK and NF-kappaB Pathways. Molecules.

[62] Waqas F.H., Shehata M., Elgaher W.A.M., Lacour A., Kurmasheva N., Begnini F., Kiib A.E., Dahlmann J., Chen C., Pavlou A. (2023). NRF2 activators inhibit influenza A virus replication by interfering with nucleo-cytoplasmic export of viral RNPs in an NRF2-independent manner. PLoS Pathog..

[63] Sethy B., Hsieh C.F., Lin T.J., Hu P.Y., Chen Y.L., Lin C.Y., Tseng S.N., Horng J.T., Hsieh P.W. (2019). Design, Synthesis, and Biological Evaluation of Itaconic Acid Derivatives as Potential Anti-Influenza Agents. J. Med. Chem..

[64] Liu Q., Gao Y., Ci X. (2019). Role of Nrf2 and Its Activators in Respiratory Diseases. Oxidative Med. Cell. Longev..

[65] Kesic M.J., Simmons S.O., Bauer R., Jaspers I. (2011). Nrf2 expression modifies influenza A entry and replication in nasal epithelial cells. Free Radic. Biol. Med..

[66] De Angelis M., Amatore D., Checconi P., Zevini A., Fraternale A., Magnani M., Hiscott J., De Chiara G., Palamara A.T., Nencioni L. (2021). Influenza Virus Down-Modulates G6PD Expression and Activity to Induce Oxidative Stress and Promote Its Replication. Front. Cell Infect. Microbiol..

[67] Zevini A., Ferrari M., Olagnier D., Hiscott J. (2020). Dengue virus infection and Nrf2 regulation of oxidative stress. Curr. Opin. Virol..

[68] Pillai A.B., Muthuraman K.R., Mariappan V., Belur S.S., Lokesh S., Rajendiran S. (2019). Oxidative stress response in the pathogenesis of dengue virus virulence, disease prognosis and therapeutics: An update. Arch. Virol..

[69] Wang J., Chen Y., Gao N., Wang Y., Tian Y., Wu J., Zhang J., Zhu J., Fan D., An J. (2013). Inhibitory effect of glutathione on oxidative liver injury induced by dengue virus serotype 2 infections in mice. PLoS ONE.

[70] Olagnier D., Peri S., Steel C., van Montfoort N., Chiang C., Beljanski V., Slifker M., He Z., Nichols C.N., Lin R. (2014). Cellular oxidative stress response controls the antiviral and apoptotic programs in dengue virus-infected dendritic cells. PLoS Pathog..

[71] Cheng Y.L., Lin Y.S., Chen C.L., Tsai T.T., Tsai C.C., Wu Y.W., Ou Y.D., Chu Y.Y., Wang J.M., Yu C.Y. (2016). Activation of Nrf2 by the dengue virus causes an increase in CLEC5A, which enhances TNF-alpha production by mononuclear phagocytes. Sci. Rep..

[72] Huang H., Falgout B., Takeda K., Yamada K.M., Dhawan S. (2017). Nrf2-dependent induction of innate host defense via heme oxygenase-1 inhibits Zika virus replication. Virology.

[73] Daskou M., Fotooh Abadi L., Gain C., Wong M., Sharma E., Kombe Kombe A.J., Nanduri R., Kelesidis T. (2023). The Role of the NRF2 Pathway in the Pathogenesis of Viral Respiratory Infections. Pathogens.

[74] Hosakote Y.M., Liu T., Castro S.M., Garofalo R.P., Casola A. (2009). Respiratory syncytial virus induces oxidative stress by modulating antioxidant enzymes. Am. J. Respir. Cell Mol. Biol..

[75] Espinoza J.A., Leon M.A., Cespedes P.F., Gomez R.S., Canedo-Marroquin G., Riquelme S.A., Salazar-Echegarai F.J., Blancou P., Simon T., Anegon I. (2017). Heme Oxygenase-1 Modulates Human Respiratory Syncytial Virus Replication and Lung Pathogenesis during Infection. J. Immunol..

[76] Casola A., Burger N., Liu T., Jamaluddin M., Brasier A.R., Garofalo R.P. (2001). Oxidant tone regulates RANTES gene expression in airway epithelial cells infected with respiratory syncytial virus. Role in viral-induced interferon regulatory factor activation. J. Biol. Chem..

[77] Shiba M., Kato T., Seko Y., Minamino-Muta E., Tanada Y., Kimura T., Ono K. (2024). Cobalt protoporphyrin promotes heme oxygenase 1 expression and ameliorates cardiac dysfunction in long-term fasting mice. Int. J. Cardiol..

[78] Sun T., Yu H.Y., Zhang C.L., Zhu T.N., Huang S.H. (2018). Respiratory syncytial virus infection up-regulates TLR7 expression by inducing oxidative stress via the Nrf2/ARE pathway in A549 cells. Arch. Virol..

[79] Cho H.Y., Imani F., Miller-DeGraff L., Walters D., Melendi G.A., Yamamoto M., Polack F.P., Kleeberger S.R. (2009). Antiviral activity of Nrf2 in a murine model of respiratory syncytial virus disease. Am. J. Respir. Crit. Care Med..

[80] Qu Y., de Mello A.H., Morris D.R., Jones-Hall Y.L., Ivanciuc T., Sattler R.A., Paessler S., Menachery V.D., Garofalo R.P., Casola A. (2023). SARS-CoV-2 Inhibits NRF2-Mediated Antioxidant Responses in Airway Epithelial Cells and in the Lung of a Murine Model of Infection. Microbiol. Spectr..

[81] Waqas F.H., Zapatero-Belinchón F.J., Carter-Timofte M.E., Lasswitz L., van der Horst D., Möller R., Dahlmann J., Olmer R., Geffers R., Gerold G. (2025). NRF2 activators restrict coronaviruses by targeting a network involving ACE2, TMPRSS2, and XPO1. bioRxiv.

[82] Sun Q., Ye F., Liang H., Liu H., Li C., Lu R., Huang B., Zhao L., Tan W., Lai L. (2021). Bardoxolone and bardoxolone methyl, two Nrf2 activators in clinical trials, inhibit SARS-CoV-2 replication and its 3C-like protease. Signal Transduct. Target. Ther..

[83] Olagnier D., Farahani E., Thyrsted J., Blay-Cadanet J., Herengt A., Idorn M., Hait A., Hernaez B., Knudsen A., Iversen M.B. (2020). SARS-CoV-2-mediated suppression of NRF2-signaling reveals potent antiviral and anti-inflammatory activity of 4-octyl-itaconate and dimethyl fumarate. Nat. Commun..

[84] Liu L., Du J., Yang S., Zheng B., Shen J., Huang J., Cao L., Huang S., Liu X., Guo L. (2023). SARS-CoV-2 ORF3a sensitizes cells to ferroptosis via Keap1-NRF2 axis. Redox Biol..

[85] De Angelis M., Anichini G., Palamara A.T., Nencioni L., Gori Savellini G. (2023). Dysregulation of intracellular redox homeostasis by the SARS-CoV-2 ORF6 protein. Virol. J..

[86] Buckley S., Byrnes S., Cochrane C., Roche M., Estes J.D., Selemidis S., Angelovich T.A., Churchill M.J. (2021). The role of oxidative stress in HIV-associated neurocognitive disorders. Brain Behav. Immun. Health.

[87] Ivanov A.V., Valuev-Elliston V.T., Ivanova O.N., Kochetkov S.N., Starodubova E.S., Bartosch B., Isaguliants M.G. (2016). Oxidative Stress during HIV Infection: Mechanisms and Consequences. Oxid. Med. Cell Longev..

[88] Israel N., Gougerot-Pocidalo M.A. (1997). Oxidative stress in human immunodeficiency virus infection. Cell Mol. Life Sci..

[89] Margaritis M. (2019). Endothelial dysfunction in HIV infection: Experimental and clinical evidence on the role of oxidative stress. Ann. Res. Hosp..

[90] Mastrantonio R., Cervelli M., Pietropaoli S., Mariottini P., Colasanti M., Persichini T. (2016). HIV-Tat Induces the Nrf2/ARE Pathway through NMDA Receptor-Elicited Spermine Oxidase Activation in Human Neuroblastoma Cells. PLoS ONE.

[91] Staitieh B.S., Ding L., Neveu W.A., Spearman P., Guidot D.M., Fan X. (2017). HIV-1 decreases Nrf2/ARE activity and phagocytic function in alveolar macrophages. J. Leukoc. Biol..

[92] Han D., Lu X., Yin W., Fu H., Zhang X., Cheng L., Liu F., Jin C., Tian X., Xie Y. (2023). Activation of NRF2 blocks HIV replication and apoptosis in macrophages. Heliyon.

[93] Ivanov A.V., Smirnova O.A., Ivanova O.N., Masalova O.V., Kochetkov S.N., Isaguliants M.G. (2011). Hepatitis C virus proteins activate NRF2/ARE pathway by distinct ROS-dependent and independent mechanisms in HUH7 cells. PLoS ONE.

[94] Sugiyama K., Ebinuma H., Nakamoto N., Sakasegawa N., Murakami Y., Chu P.S., Usui S., Ishibashi Y., Wakayama Y., Taniki N. (2014). Prominent steatosis with hypermetabolism of the cell line permissive for years of infection with hepatitis C virus. PLoS ONE.

[95] Medvedev R., Ploen D., Spengler C., Elgner F., Ren H., Bunten S., Hildt E. (2017). HCV-induced oxidative stress by inhibition of Nrf2 triggers autophagy and favors release of viral particles. Free Radic. Biol. Med..

[96] Chu J.Y.K., Ou J.J. (2021). Autophagy in HCV Replication and Protein Trafficking. Int. J. Mol. Sci..

[97] Carvajal-Yepes M., Himmelsbach K., Schaedler S., Ploen D., Krause J., Ludwig L., Weiss T., Klingel K., Hildt E. (2011). Hepatitis C virus impairs the induction of cytoprotective Nrf2 target genes by delocalization of small Maf proteins. J. Biol. Chem..

[98] Schaedler S., Krause J., Himmelsbach K., Carvajal-Yepes M., Lieder F., Klingel K., Nassal M., Weiss T.S., Werner S., Hildt E. (2010). Hepatitis B virus induces expression of antioxidant response element-regulated genes by activation of Nrf2. J. Biol. Chem..

[99] Taira J., Kubo T., Nagano H., Tsuda R., Ogi T., Nakashima K., Suzuki T. (2025). Effect of Nrf2 Activators in Hepatitis B Virus-Infected Cells Under Oxidative Stress. Mar. Drugs.

[100] Liu B., Fang M., He Z., Cui D., Jia S., Lin X., Xu X., Zhou T., Liu W. (2015). Hepatitis B virus stimulates G6PD expression through HBx-mediated Nrf2 activation. Cell Death Dis..

[101] Wyler E., Franke V., Menegatti J., Kocks C., Boltengagen A., Praktiknjo S., Walch-Ruckheim B., Bosse J., Rajewsky N., Grasser F. (2019). Single-cell RNA-sequencing of herpes simplex virus 1-infected cells connects NRF2 activation to an antiviral program. Nat. Commun..

[102] Zhang L., Wang J., Wang Z., Li Y., Wang H., Liu H. (2022). Upregulation of nuclear factor E2-related factor 2 (Nrf2) represses the replication of herpes simplex virus type 1. Virol. J..

[103] Kim B., Kim Y.S., Li W., Kwon E.B., Chung H.S., Go Y., Choi J.G. (2024). Ginsenoside Rg5, a potent agonist of Nrf2, inhibits HSV-1 infection-induced neuroinflammation by inhibiting oxidative stress and NF-kappaB activation. J. Ginseng Res..

[104] Seelige R., Saddawi-Konefka R., Adams N.M., Picarda G., Sun J.C., Benedict C.A., Bui J.D. (2018). Interleukin-17D and Nrf2 mediate initial innate immune cell recruitment and restrict MCMV infection. Sci. Rep..

[105] Lee J., Koh K., Kim Y.E., Ahn J.H., Kim S. (2013). Upregulation of Nrf2 expression by human cytomegalovirus infection protects host cells from oxidative stress. J. Gen. Virol..

[106] Mills E.L., Ryan D.G., Prag H.A., Dikovskaya D., Menon D., Zaslona Z., Jedrychowski M.P., Costa A.S.H., Higgins M., Hams E. (2018). Itaconate is an anti-inflammatory metabolite that activates Nrf2 via alkylation of KEAP1. Nature.

[107] Olagnier D., Brandtoft A.M., Gunderstofte C., Villadsen N.L., Krapp C., Thielke A.L., Laustsen A., Peri S., Hansen A.L., Bonefeld L. (2018). Nrf2 negatively regulates STING indicating a link between antiviral sensing and metabolic reprogramming. Nat. Commun..

[108] Kurmasheva N., Said A., Wong B., Kinderman P., Han X., Rahimic A.H.F., Kress A., Carter-Timofte M.E., Holm E., van der Horst D. (2024). Octyl itaconate enhances VSVDelta51 oncolytic virotherapy by multitarget inhibition of antiviral and inflammatory pathways. Nat. Commun..

[109] Isaguliants M., Smirnova O., Ivanov A.V., Kilpelainen A., Kuzmenko Y., Petkov S., Latanova A., Krotova O., Engstrom G., Karpov V. (2013). Oxidative stress induced by HIV-1 reverse transcriptase modulates the enzyme’s performance in gene immunization. Hum. Vaccines Immunother..

[110] Liu P., Wang X., Sun Y., Zhao H., Cheng F., Wang J., Yang F., Hu J., Zhang H., Wang C.-C. (2022). SARS-CoV-2 ORF8 reshapes the ER through forming mixed disulfides with ER oxidoreductases. Redox Biol..

[111] Cullinan S.B., Zhang D., Hannink M., Arvisais E., Kaufman R.J., Diehl J.A. (2003). Nrf2 is a direct PERK substrate and effector of PERK-dependent cell survival. Mol. Cell Biol..

[112] Saito T., Ichimura Y., Taguchi K., Suzuki T., Mizushima T., Takagi K., Hirose Y., Nagahashi M., Iso T., Fukutomi T. (2016). p62/Sqstm1 promotes malignancy of HCV-positive hepatocellular carcinoma through Nrf2-dependent metabolic reprogramming. Nat. Commun..

[113] Hu Z., Zhang Z., Doo E., Coux O., Goldberg A.L., Liang T.J. (1999). Hepatitis B Virus X Protein Is both a Substrate and a Potential Inhibitor of the Proteasome Complex. J. Virol..

[114] Sapochnik D., Raimondi A.R., Medina V., Naipauer J., Mesri E.A., Coso O. (2022). A major role for Nrf2 transcription factors in cell transformation by KSHV encoded oncogenes. Front. Oncol..

[115] Robledinos-Anton N., Fernandez-Gines R., Manda G., Cuadrado A. (2019). Activators and Inhibitors of NRF2: A Review of Their Potential for Clinical Development. Oxid. Med. Cell Longev..

[116] Satoh T., McKercher S.R., Lipton S.A. (2013). Nrf2/ARE-mediated antioxidant actions of pro-electrophilic drugs. Free Radic. Biol. Med..

[117] Noah T.L., Zhang H., Zhou H., Glista-Baker E., Muller L., Bauer R.N., Meyer M., Murphy P.C., Jones S., Letang B. (2014). Effect of broccoli sprouts on nasal response to live attenuated influenza virus in smokers: A randomized, double-blind study. PLoS ONE.

[118] Cho W.K., Yim N.H., Lee M.M., Han C.H., Ma J.Y. (2022). Broccoli Leaves Attenuate Influenza A Virus Infection by Interfering With Hemagglutinin and Inhibiting Viral Attachment. Front. Pharmacol..

[119] Yageta Y., Ishii Y., Morishima Y., Ano S., Ohtsuka S., Matsuyama M., Takeuchi K., Itoh K., Yamamoto M., Hizawa N. (2014). Carbocisteine reduces virus-induced pulmonary inflammation in mice exposed to cigarette smoke. Am. J. Respir. Cell Mol. Biol..

[120] McAuley J.L., Tate M.D., MacKenzie-Kludas C.J., Pinar A., Zeng W., Stutz A., Latz E., Brown L.E., Mansell A. (2013). Activation of the NLRP3 inflammasome by IAV virulence protein PB1-F2 contributes to severe pathophysiology and disease. PLoS Pathog..

[121] Dai J., Gu L., Su Y., Wang Q., Zhao Y., Chen X., Deng H., Li W., Wang G., Li K. (2018). Inhibition of curcumin on influenza A virus infection and influenzal pneumonia via oxidative stress, TLR2/4, p38/JNK MAPK and NF-kappaB pathways. Int. Immunopharmacol..

[122] Zhang C., Zhang K., Zang G., Chen T., Lu N., Wang S., Zhang G. (2021). Curcumin Inhibits Replication of Human Parainfluenza Virus Type 3 by Affecting Viral Inclusion Body Formation. Biomed. Res. Int..

[123] Chen T.Y., Chen D.Y., Wen H.W., Ou J.L., Chiou S.S., Chen J.M., Wong M.L., Hsu W.L. (2013). Inhibition of enveloped viruses infectivity by curcumin. PLoS ONE.

[124] Nizam N.N., Mahmud S., Ark S.M.A., Kamruzzaman M., Hasan M.K. (2023). Bakuchiol, a natural constituent and its pharmacological benefits. F1000Research.

[125] Shoji M., Arakaki Y., Esumi T., Kohnomi S., Yamamoto C., Suzuki Y., Takahashi E., Konishi S., Kido H., Kuzuhara T. (2015). Bakuchiol Is a Phenolic Isoprenoid with Novel Enantiomer-selective Anti-influenza A Virus Activity Involving Nrf2 Activation. J. Biol. Chem..

[126] Inoyama D., Chen Y., Huang X., Beamer L.J., Kong A.N., Hu L. (2012). Optimization of fluorescently labeled Nrf2 peptide probes and the development of a fluorescence polarization assay for the discovery of inhibitors of Keap1-Nrf2 interaction. J. Biomol. Screen..

[127] Hancock R., Schaap M., Pfister H., Wells G. (2013). Peptide inhibitors of the Keap1-Nrf2 protein-protein interaction with improved binding and cellular activity. Org. Biomol. Chem..

[128] Richardson B.G., Jain A.D., Speltz T.E., Moore T.W. (2015). Non-electrophilic modulators of the canonical Keap1/Nrf2 pathway. Bioorganic Med. Chem. Lett..

[129] Qin Y., Poulsen C., Narayanan D., Chan C.B., Chen X., Montes B.R., Tran K.T., Mukminova E., Lin C., Gajhede M. (2024). Structure-Guided Conformational Restriction Leading to High-Affinity, Selective, and Cell-Active Tetrahydroisoquinoline-Based Noncovalent Keap1-Nrf2 Inhibitors. J. Med. Chem..

[130] Tran K.T., Pallesen J.S., Solbak S.M.O., Narayanan D., Baig A., Zang J., Aguayo-Orozco A., Carmona R.M.C., Garcia A.D., Bach A. (2019). A Comparative Assessment Study of Known Small-Molecule Keap1-Nrf2 Protein-Protein Interaction Inhibitors: Chemical Synthesis, Binding Properties, and Cellular Activity. J. Med. Chem..

[131] Barreca M., Qin Y., Cadot M.E.H., Barraja P., Bach A. (2023). Advances in developing noncovalent small molecules targeting Keap1. Drug Discov. Today.

[132] Ge Y., Zhang C., Qu Y., Ding L., Zhang X., Zhang Z., Jin C., Wang X.N., Wang Z. (2023). Synthesis and pharmacodynamic evaluation of naphthalene derivatives against influenza A virus in vitro and in vivo. Eur. J. Med. Chem..

